# 1-Eth­oxy-3-[4-(eth­oxy­carbon­yl)phen­yl]-3-hy­droxy-1-oxopropan-2-aminium chloride

**DOI:** 10.1107/S2414314624010320

**Published:** 2024-10-31

**Authors:** Dieter Schollmeyer, Igor Proz, Heiner Detert

**Affiliations:** a University of Mainz, Department of Chemistry, Duesbergweg 10-14, 55099 Mainz, Germany; Goethe-Universität Frankfurt, Germany

**Keywords:** crystal structure, amino acid, racemate, amino­alcohol, phenyl alanine

## Abstract

The title compound, C_14_H_20_NO_5_^+^·Cl^−^, was prepared as a racemate of *R*,*R*- and *S*,*S*-enanti­omers by reduction of the corresponding hy­droxy­imino­ketone. In the crystal, layers are formed *via* hydrogen bridges of four ammonium groups to chloride ions; these lamellae are connected *via* inter­digitated benzoic ester groups.

## Abstract 2

The title compound was prepared as a racemate of *R*,*R*- and *S*,*S*-enanti­omers by reduction of the corresponding hy­droxy­imino­ketone. In the crystal, layers are formed *via* hydrogen bridges of four ammonium groups to chloride ions; these lamellae are connected *via* inter­digitated benzoic ester groups.

## Graphical abstract

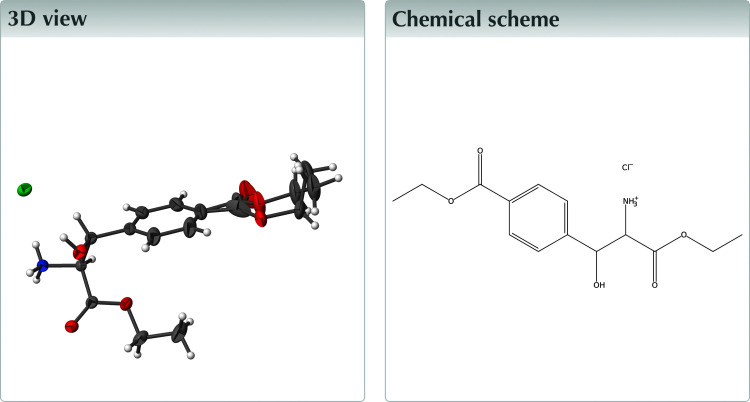

## Structure description

**Figure 1:**
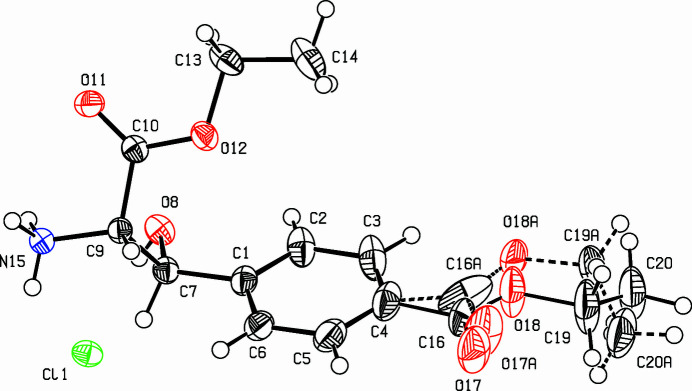
View of the title compound. Displacement ellipsoids are drawn at the 50% probability level. The bonds involving the minor occupied sites are drawn as broken bonds.

**Figure 2:**
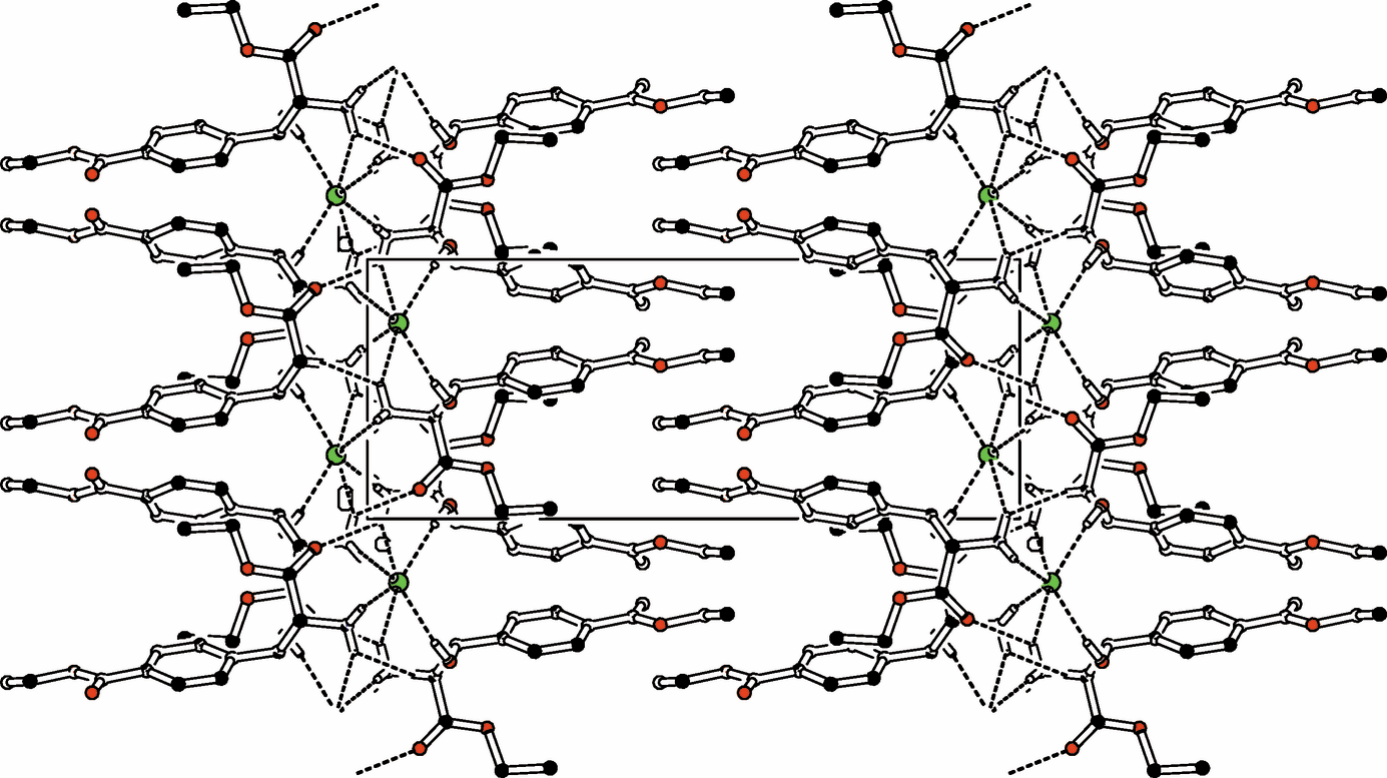
Part of the packing diagram. View along the *c* axis.

The title compound, [C_14_H_20_NO_5_]^+^·Cl^−^ (Fig. 1 ▸), was prepared by nitro­sation of the corres­ponding keto-ester followed by catalytic reduction of the resulting hy­droxy­imino­ketone. As was observed with a similar oxime (Ebel & Deuschel, 1956 ▸), the hydrogenation occurred on both functional groups, the oxime and the ketone, generating two vicinal chiral centers. The crystalline compound obtained from the hydrogenation is a racemic mixture of the (*S*,*S*)- and (*R*,*R*)-enanti­omers. The compound crystallizes with a disordered benzoic ester function, the ratio of the two conformers being 0.745/0.255 (11). The minor occupied conformer exhibits a dihedral angle between the plane of the benzene ring and the ester function (C16*A*—O17*A*—O18*A*—C19*A*—C20*A*) of 29.8 (9)°, whereas in the major occupied conformer, the planes of the ring and the ethyl ester (C16—O17—O18—C19—C20) subtend an angle of 14.0 (3)°, with a maximum deviation of −0.148 (7) Å from the ester mean plane at C19. The other ester moiety (C9—C10—O11—O12—C13—C14) is also nearly planar [maximum deviation from mean plane on C14: 0.0733 (19) Å]. This plane and the benzene ring subtend a dihedral angle of 49.16 (8)°. The hy­droxy and amino groups are nearly staggered, the torsion angle O8—C7—C9—N15 being 71.81 (13)°. All the ammonium hydrogen atoms are connected with symmetry-related chloride ions *via* N—H⋯Cl hydrogen bonds (Fig. 2 ▸, Table 1 ▸); furthermore, H15*A* forms an inter­molecular bond to the carbonyl oxygen O1, therefore, four mol­ecules are connected *via* hydrogen bonds to one chloride ion and a carbonyl O atom. The packing in the crystal is dominated by the hydrogen bridges, resulting in lamellae formation in the *bc* plane. These layers are connected in the *ac* plane *via* inter­digitated benzoic ester groups, connected *via* van der Waals inter­actions.

## Synthesis and crystallization

Ethyl 4-(3-eth­oxy-3-oxo­propano­yl)benzoate (Korsager *et al.*, 2013 ▸) (1.95 g, 7.4 mmol) was added to glacial acetic acid (1.8 ml) at 283 K. While stirring, a solution of 0.76 g NaNO_2_ (11 mmol) in a minimal amount of water was added dropwise. The mixture was allowed to reach room temperature and after stirring for 1 h, the product was extracted with ethyl acetate, pooled extracts were deacidified with sodium bicarbonate, dried, and the product, ethyl 4-(3-eth­oxy-2-hy­droxy­imino-3-oxo­propano­yl)benzoate, was purified by column chromatography (SiO_2_, petroleum ether/ethyl acetate 20/1, then toluene /ethyl acetate 9/1) colorless oil, yield: 1.43 g, 66%. 200 mg (0.7 mmol) of this compound were dissolved in ethanol (1 ml) and Pd/C (5%, 0.24 g) and hydro­chloric acid (36%, 0.05 ml) were added, stirring for 48 h in a hydrogen atmos­phere. The mixture was filtered through silica, the silica washed with ethanol and the solvent was slowly evaporated to obtain 63 mg (0.2 mmol, 32%) as a colorless solid with m.p.= 445-447 K. ^1^H-NMR (400 MHz, D_2_O): 7.97 (*m*, 2 H), 7.43 (*d*, *J* = 8.3 Hz, 2 H), 5.39 (*d*, 1 H), 4.44 (*q*, 1 H), 4.29 (*m*, *J* = 7.1 Hz, 2 H), 4.04 (*qd*, *J* = 7.2 Hz, 2 H), 1.28 (*t*, *J* = 7.1 Hz, 3 H), 0.97 (*t*, *J* = 7.1 Hz, 3 H). C-NMR (100 MHz, D_2_O): 168.52, 167.16, 143.05, 130.05, 129.68, 126.07, 70.57, 63.29, 62,34, 58.16, 13.34, 12.89. IR: 2985, 2356, 1718, 1506, 1370, 1277, 1107, 1017, 960, 868, 862, 821, 805 cm^−1^. MS (ESI) 282.13.

## Refinement

Crystal data, data collection and structure refinement details are summarized in Table 2 ▸.

## Supplementary Material

Crystal structure: contains datablock(s) I, global. DOI: 10.1107/S2414314624010320/bt4157sup1.cif

Structure factors: contains datablock(s) I. DOI: 10.1107/S2414314624010320/bt4157Isup2.hkl

Supporting information file. DOI: 10.1107/S2414314624010320/bt4157Isup3.cml

CCDC reference: 2393082

Additional supporting information:  crystallographic information; 3D view; checkCIF report

## Figures and Tables

**Table 1:** Hydrogen-bond geometry (Å, °)

*D*—H⋯*A*	*D*—H	H⋯*A*	*D*⋯*A*	*D*—H⋯*A*
C7—H7⋯Cl1	1.00	2.89	3.4784 (13)	119
C7—H7⋯Cl1^i^	1.00	2.96	3.6365 (14)	126
O8—H8⋯Cl1	0.84 (2)	2.24 (2)	3.0625 (11)	165.1 (19)
N15—H15*A*⋯Cl1^i^	0.87 (2)	2.53 (2)	3.2470 (13)	140.7 (16)
N15—H15*A*⋯O11^ii^	0.87 (2)	2.279 (19)	2.8484 (15)	123.0 (16)
N15—H15*B*⋯Cl1^iii^	0.94 (2)	2.31 (2)	3.2011 (12)	159.3 (15)
N15—H15*C*⋯Cl1^iv^	0.94 (2)	2.19 (2)	3.1155 (13)	168.4 (16)
C20—H20*A*⋯O17^v^	0.98	2.64	3.520 (6)	149
C20*A*—H20*D*⋯O18*A*^vi^	0.98	2.03	2.75 (2)	129

Symmetry codes: (i) 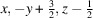; (ii) 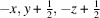; (iii) 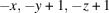; (iv) 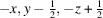; (v) 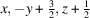; (vi) 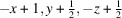.

**Table 2:** Experimental details

Crystal data	
Chemical formula	C_14_H_20_NO_5_^+^·Cl^−^
*M*_r_	317.76
Crystal system, space group	Monoclinic, *P*2_1_/*c*
Temperature (K)	120
*a*, *b*, *c* (Å)	20.5205 (9), 8.1626 (3), 9.4732 (4)
β (°)	90.788 (4)
*V* (Å^3^)	1586.62 (11)
*Z*	4
Radiation type	Mo *K*α
μ (mm^−1^)	0.26
Crystal size (mm)	0.45 × 0.26 × 0.07

Data collection	
Diffractometer	Stoe *IPDS* 2T
No. of measured, independent and observed [*I* > 2σ(*I*)] reflections	13552, 3856, 3370
*R*_int_	0.025
(sin θ/λ)_max_ (Å^−1^)	0.663

Refinement	
*R*[*F*^2^ > 2σ(*F*^2^)], *wR*(*F*^2^), *S*	0.038, 0.100, 1.07
No. of reflections	3856
No. of parameters	253
H-atom treatment	H atoms treated by a mixture of independent and constrained refinement
Δρ_max_, Δρ_min_ (e Å^−3^)	0.37, −0.24

Computer programs: *X-AREA WinXpose*, * Recipe* and *Integrate* (Stoe & Cie, 2020 ▸), *SHELXT2014* (Sheldrick, 2015*a* ▸), *SHELXL2019/2* (Sheldrick, 2015*b* ▸) and *PLATON* (Spek, 2020 ▸).

## full crystallographic data

### 1-Ethoxy-3-[4-(ethoxycarbonyl)phenyl]-3-hydroxy-1-oxopropan-2-aminium chloride Crystal data

**Table:**

C_14_H_20_NO_5_^+^·Cl^−^	*F*(000) = 672
*M**_r_* = 317.76	*D*_x_ = 1.330 Mg m^−^^3^
Monoclinic, *P*2_1_/*c*	Mo *K*α radiation, λ = 0.71073 Å
*a* = 20.5205 (9) Å	Cell parameters from 20325 reflections
*b* = 8.1626 (3) Å	θ = 2.7–28.6°
*c* = 9.4732 (4) Å	µ = 0.26 mm^−^^1^
β = 90.788 (4)°	*T* = 120 K
*V* = 1586.62 (11) Å^3^	Plate, colorless
*Z* = 4	0.45 × 0.26 × 0.07 mm

### 1-Ethoxy-3-[4-(ethoxycarbonyl)phenyl]-3-hydroxy-1-oxopropan-2-aminium chloride Data collection

**Table:**

Stoe IPDS 2T diffractometer	3370 reflections with *I* > 2σ(*I*)
Radiation source: sealed X-ray tube, 12x0.4mm long-fine focus	*R*_int_ = 0.025
Detector resolution: 6.67 pixels mm^-1^	θ_max_ = 28.1°, θ_min_ = 2.7°
rotation method, ω scans	*h* = −26→27
13552 measured reflections	*k* = −10→10
3856 independent reflections	*l* = −12→12

### 1-Ethoxy-3-[4-(ethoxycarbonyl)phenyl]-3-hydroxy-1-oxopropan-2-aminium chloride Refinement

**Table:**

Refinement on *F*^2^	Primary atom site location: dual
Least-squares matrix: full	Hydrogen site location: mixed
*R*[*F*^2^ > 2σ(*F*^2^)] = 0.038	H atoms treated by a mixture of independent and constrained refinement
*wR*(*F*^2^) = 0.100	*w* = 1/[σ^2^(*F*_o_^2^) + (0.0426*P*)^2^ + 0.8357*P*] where *P* = (*F*_o_^2^ + 2*F*_c_^2^)/3
*S* = 1.07	(Δ/σ)_max_ = 0.001
3856 reflections	Δρ_max_ = 0.37 e Å^−^^3^
253 parameters	Δρ_min_ = −0.24 e Å^−^^3^
0 restraints	

### 1-Ethoxy-3-[4-(ethoxycarbonyl)phenyl]-3-hydroxy-1-oxopropan-2-aminium chloride Special details

**Table:**

Geometry. All esds (except the esd in the dihedral angle between two l.s. planes) are estimated using the full covariance matrix. The cell esds are taken into account individually in the estimation of esds in distances, angles and torsion angles; correlations between esds in cell parameters are only used when they are defined by crystal symmetry. An approximate (isotropic) treatment of cell esds is used for estimating esds involving l.s. planes.
Refinement. Hydrogen atoms attached to carbons were placed at calculated positions and were refined in the riding-model approximation with C_aromatic_–H = 0.95 Å, C_methyl_–H = 0.98 Å, C_methylene_–H = 0.99 Å, C_tertiary_–H = 1.00 Å, and with *U*_iso_(H) = 1.5 *U*_eq_(C_methyl_) or with *U*_iso_(H) = 1.2 *U*_eq_(C). The methyl groups were allowed to rotate but not to tip. The H atom bonded to O was freely refined. The coordinates of the H atoms bonded to N were freely refined, using the same displacement parameter for all of them.

### 1-Ethoxy-3-[4-(ethoxycarbonyl)phenyl]-3-hydroxy-1-oxopropan-2-aminium chloride Fractional atomic coordinates and isotropic or equivalent isotropic displacement parameters (Å^2^)

**Table:**

	*x*	*y*	*z*	*U*_iso_*/*U*_eq_	Occ. (<1)
Cl1	0.04788 (2)	0.74584 (4)	0.53772 (3)	0.02717 (10)	
C1	0.20763 (7)	0.53804 (17)	0.26788 (16)	0.0290 (3)	
C2	0.25645 (7)	0.4881 (2)	0.36094 (18)	0.0396 (4)	
H2	0.245492	0.436695	0.447396	0.047*	
C3	0.32166 (8)	0.5135 (3)	0.3275 (2)	0.0489 (5)	
H3	0.354962	0.480069	0.391941	0.059*	
C4	0.33832 (8)	0.5868 (2)	0.2013 (2)	0.0459 (4)	
C5	0.28952 (8)	0.6384 (2)	0.1090 (2)	0.0432 (4)	
H5	0.300619	0.689710	0.022632	0.052*	
C6	0.22461 (8)	0.61534 (19)	0.14216 (18)	0.0356 (3)	
H6	0.191439	0.652400	0.078923	0.043*	
C7	0.13615 (6)	0.51234 (17)	0.29996 (14)	0.0247 (3)	
H7	0.113579	0.620639	0.292571	0.030*	
O8	0.12629 (5)	0.44954 (13)	0.43765 (10)	0.0290 (2)	
H8	0.1037 (10)	0.520 (3)	0.479 (2)	0.044 (5)*	
C9	0.10290 (6)	0.39315 (16)	0.19502 (14)	0.0224 (3)	
H9	0.116469	0.419779	0.096663	0.027*	
C10	0.11986 (6)	0.21598 (16)	0.22877 (14)	0.0242 (3)	
O11	0.08032 (5)	0.11362 (12)	0.25813 (12)	0.0318 (2)	
O12	0.18364 (5)	0.19266 (13)	0.22279 (11)	0.0302 (2)	
C13	0.20667 (8)	0.0287 (2)	0.25851 (19)	0.0387 (4)	
H13A	0.192695	−0.050854	0.185213	0.046*	
H13B	0.188989	−0.006596	0.350352	0.046*	
C14	0.27998 (9)	0.0386 (3)	0.2664 (2)	0.0529 (5)	
H14A	0.297970	−0.070463	0.285645	0.079*	
H14B	0.293063	0.113733	0.342381	0.079*	
H14C	0.296576	0.079071	0.176389	0.079*	
N15	0.03125 (5)	0.41088 (15)	0.20662 (13)	0.0244 (2)	
H15A	0.0204 (9)	0.513 (3)	0.193 (2)	0.037 (3)*	
H15B	0.0158 (9)	0.381 (2)	0.296 (2)	0.037 (3)*	
H15C	0.0093 (9)	0.347 (2)	0.139 (2)	0.037 (3)*	
C16	0.40734 (16)	0.6211 (5)	0.1514 (7)	0.0389 (10)	0.745 (11)
O17	0.42199 (18)	0.6711 (5)	0.0352 (5)	0.0582 (9)	0.745 (11)
O18	0.44929 (13)	0.5912 (7)	0.2542 (4)	0.0615 (10)	0.745 (11)
C19	0.51701 (16)	0.6271 (9)	0.2243 (5)	0.0714 (15)	0.745 (11)
H19A	0.520857	0.734003	0.175759	0.086*	0.745 (11)
H19B	0.535769	0.541044	0.163486	0.086*	0.745 (11)
C20	0.55162 (16)	0.6316 (9)	0.3649 (5)	0.0711 (13)	0.745 (11)
H20A	0.530360	0.711548	0.426259	0.085*	0.745 (11)
H20B	0.597186	0.663354	0.351674	0.085*	0.745 (11)
H20C	0.549900	0.522924	0.408636	0.085*	0.745 (11)
C16A	0.4092 (10)	0.583 (2)	0.2027 (17)	0.062 (5)	0.255 (11)
O17A	0.4281 (7)	0.6462 (18)	0.0916 (14)	0.070 (3)	0.255 (11)
O18A	0.4507 (3)	0.5225 (12)	0.2963 (14)	0.049 (2)	0.255 (11)
C19A	0.5220 (4)	0.5443 (15)	0.2706 (19)	0.061 (4)	0.255 (11)
H19C	0.547117	0.463037	0.326574	0.073*	0.255 (11)
H19D	0.531053	0.525759	0.169490	0.073*	0.255 (11)
C20A	0.5421 (5)	0.710 (2)	0.311 (2)	0.069 (4)	0.255 (11)
H20D	0.521517	0.789492	0.246725	0.103*	0.255 (11)
H20E	0.589582	0.719229	0.304779	0.103*	0.255 (11)
H20F	0.528635	0.732273	0.407778	0.103*	0.255 (11)

### 1-Ethoxy-3-[4-(ethoxycarbonyl)phenyl]-3-hydroxy-1-oxopropan-2-aminium chloride Atomic displacement parameters (Å^2^)

**Table:**

	*U*^11^	*U*^22^	*U*^33^	*U*^12^	*U*^13^	*U*^23^
Cl1	0.03246 (18)	0.02436 (17)	0.02476 (16)	0.00383 (12)	0.00350 (12)	−0.00027 (12)
C1	0.0241 (6)	0.0261 (7)	0.0367 (7)	−0.0015 (5)	0.0013 (5)	−0.0113 (6)
C2	0.0272 (7)	0.0524 (10)	0.0391 (8)	0.0013 (7)	−0.0027 (6)	−0.0125 (7)
C3	0.0260 (8)	0.0657 (12)	0.0549 (11)	0.0042 (8)	−0.0059 (7)	−0.0210 (9)
C4	0.0282 (8)	0.0449 (9)	0.0649 (12)	−0.0056 (7)	0.0105 (7)	−0.0211 (9)
C5	0.0369 (8)	0.0345 (8)	0.0585 (11)	−0.0070 (7)	0.0125 (7)	−0.0056 (8)
C6	0.0305 (7)	0.0286 (7)	0.0479 (9)	−0.0028 (6)	0.0046 (6)	−0.0040 (6)
C7	0.0235 (6)	0.0248 (6)	0.0257 (6)	0.0012 (5)	0.0003 (5)	−0.0037 (5)
O8	0.0306 (5)	0.0323 (5)	0.0242 (5)	0.0066 (4)	0.0009 (4)	−0.0040 (4)
C9	0.0206 (6)	0.0227 (6)	0.0239 (6)	0.0000 (5)	0.0005 (5)	−0.0008 (5)
C10	0.0250 (6)	0.0252 (6)	0.0223 (6)	0.0026 (5)	0.0007 (5)	−0.0035 (5)
O11	0.0303 (5)	0.0238 (5)	0.0415 (6)	0.0008 (4)	0.0059 (4)	0.0021 (4)
O12	0.0250 (5)	0.0282 (5)	0.0372 (5)	0.0050 (4)	−0.0006 (4)	−0.0044 (4)
C13	0.0395 (8)	0.0339 (8)	0.0425 (9)	0.0154 (7)	−0.0041 (7)	−0.0026 (7)
C14	0.0408 (9)	0.0609 (12)	0.0566 (11)	0.0239 (9)	−0.0127 (8)	−0.0142 (9)
N15	0.0221 (5)	0.0233 (5)	0.0277 (6)	0.0006 (4)	−0.0010 (4)	0.0017 (5)
C16	0.0231 (14)	0.0503 (19)	0.044 (3)	−0.0071 (12)	0.0116 (14)	−0.0005 (17)
O17	0.0346 (11)	0.0832 (19)	0.057 (2)	−0.0068 (11)	0.0143 (15)	0.0125 (17)
O18	0.0223 (10)	0.098 (3)	0.0647 (17)	−0.0070 (14)	0.0010 (10)	0.0146 (17)
C19	0.0209 (13)	0.112 (4)	0.081 (2)	−0.0046 (19)	0.0032 (13)	0.014 (3)
C20	0.0244 (13)	0.106 (4)	0.083 (3)	−0.0029 (18)	−0.0030 (14)	0.012 (2)
C16A	0.101 (12)	0.054 (7)	0.033 (7)	−0.031 (6)	0.041 (7)	−0.003 (5)
O17A	0.054 (6)	0.113 (8)	0.044 (6)	−0.022 (5)	0.025 (5)	0.021 (5)
O18A	0.024 (3)	0.046 (4)	0.076 (6)	−0.005 (3)	0.014 (3)	0.001 (3)
C19A	0.021 (4)	0.054 (6)	0.108 (11)	0.007 (4)	0.011 (5)	−0.006 (6)
C20A	0.029 (5)	0.079 (9)	0.098 (11)	−0.013 (5)	0.015 (5)	−0.016 (7)

### 1-Ethoxy-3-[4-(ethoxycarbonyl)phenyl]-3-hydroxy-1-oxopropan-2-aminium chloride Geometric parameters (Å, º)

**Table:**

C1—C2	1.387 (2)	C13—H13B	0.9900
C1—C6	1.396 (2)	C14—H14A	0.9800
C1—C7	1.5165 (18)	C14—H14B	0.9800
C2—C3	1.395 (2)	C14—H14C	0.9800
C2—H2	0.9500	N15—H15A	0.87 (2)
C3—C4	1.384 (3)	N15—H15B	0.94 (2)
C3—H3	0.9500	N15—H15C	0.94 (2)
C4—C5	1.386 (3)	C16—O17	1.216 (5)
C4—C16A	1.45 (2)	C16—O18	1.314 (5)
C4—C16	1.525 (5)	O18—C19	1.452 (4)
C5—C6	1.385 (2)	C19—C20	1.501 (7)
C5—H5	0.9500	C19—H19A	0.9900
C6—H6	0.9500	C19—H19B	0.9900
C7—O8	1.4186 (16)	C20—H20A	0.9800
C7—C9	1.5433 (18)	C20—H20B	0.9800
C7—H7	1.0000	C20—H20C	0.9800
O8—H8	0.84 (2)	C16A—O17A	1.238 (10)
C9—N15	1.4831 (16)	C16A—O18A	1.32 (2)
C9—C10	1.5204 (18)	O18A—C19A	1.496 (11)
C9—H9	1.0000	C19A—C20A	1.463 (18)
C10—O11	1.2000 (17)	C19A—H19C	0.9900
C10—O12	1.3245 (16)	C19A—H19D	0.9900
O12—C13	1.4579 (18)	C20A—H20D	0.9800
C13—C14	1.508 (2)	C20A—H20E	0.9800
C13—H13A	0.9900	C20A—H20F	0.9800
			
C2—C1—C6	119.24 (14)	C13—C14—H14B	109.5
C2—C1—C7	121.62 (14)	H14A—C14—H14B	109.5
C6—C1—C7	119.14 (13)	C13—C14—H14C	109.5
C1—C2—C3	119.93 (17)	H14A—C14—H14C	109.5
C1—C2—H2	120.0	H14B—C14—H14C	109.5
C3—C2—H2	120.0	C9—N15—H15A	109.5 (12)
C4—C3—C2	120.63 (17)	C9—N15—H15B	112.9 (11)
C4—C3—H3	119.7	H15A—N15—H15B	107.4 (17)
C2—C3—H3	119.7	C9—N15—H15C	111.3 (11)
C3—C4—C5	119.42 (15)	H15A—N15—H15C	108.0 (17)
C3—C4—C16A	104.1 (7)	H15B—N15—H15C	107.7 (16)
C5—C4—C16A	136.5 (7)	O17—C16—O18	124.5 (3)
C3—C4—C16	126.1 (3)	O17—C16—C4	125.8 (3)
C5—C4—C16	114.5 (3)	O18—C16—C4	109.8 (4)
C6—C5—C4	120.30 (17)	C16—O18—C19	115.9 (3)
C6—C5—H5	119.8	O18—C19—C20	105.9 (3)
C4—C5—H5	119.8	O18—C19—H19A	110.6
C5—C6—C1	120.44 (16)	C20—C19—H19A	110.6
C5—C6—H6	119.8	O18—C19—H19B	110.6
C1—C6—H6	119.8	C20—C19—H19B	110.6
O8—C7—C1	112.63 (11)	H19A—C19—H19B	108.7
O8—C7—C9	107.28 (11)	C19—C20—H20A	109.5
C1—C7—C9	112.23 (11)	C19—C20—H20B	109.5
O8—C7—H7	108.2	H20A—C20—H20B	109.5
C1—C7—H7	108.2	C19—C20—H20C	109.5
C9—C7—H7	108.2	H20A—C20—H20C	109.5
C7—O8—H8	105.4 (14)	H20B—C20—H20C	109.5
N15—C9—C10	107.55 (10)	O17A—C16A—O18A	121.4 (15)
N15—C9—C7	108.70 (10)	O17A—C16A—C4	108.0 (16)
C10—C9—C7	111.49 (11)	O18A—C16A—C4	130.6 (9)
N15—C9—H9	109.7	C16A—O18A—C19A	118.1 (9)
C10—C9—H9	109.7	C20A—C19A—O18A	109.9 (8)
C7—C9—H9	109.7	C20A—C19A—H19C	109.7
O11—C10—O12	125.56 (13)	O18A—C19A—H19C	109.7
O11—C10—C9	123.88 (12)	C20A—C19A—H19D	109.7
O12—C10—C9	110.56 (11)	O18A—C19A—H19D	109.7
C10—O12—C13	116.06 (12)	H19C—C19A—H19D	108.2
O12—C13—C14	106.39 (15)	C19A—C20A—H20D	109.5
O12—C13—H13A	110.5	C19A—C20A—H20E	109.5
C14—C13—H13A	110.5	H20D—C20A—H20E	109.5
O12—C13—H13B	110.5	C19A—C20A—H20F	109.5
C14—C13—H13B	110.5	H20D—C20A—H20F	109.5
H13A—C13—H13B	108.6	H20E—C20A—H20F	109.5
C13—C14—H14A	109.5		
			
C6—C1—C2—C3	−0.8 (2)	C7—C9—C10—O11	119.45 (14)
C7—C1—C2—C3	179.81 (15)	N15—C9—C10—O12	−178.83 (11)
C1—C2—C3—C4	−0.6 (3)	C7—C9—C10—O12	−59.76 (14)
C2—C3—C4—C5	1.3 (3)	O11—C10—O12—C13	−1.5 (2)
C2—C3—C4—C16A	−177.1 (6)	C9—C10—O12—C13	177.71 (11)
C2—C3—C4—C16	−179.7 (2)	C10—O12—C13—C14	−171.43 (13)
C3—C4—C5—C6	−0.6 (3)	C3—C4—C16—O17	172.6 (4)
C16A—C4—C5—C6	177.3 (8)	C5—C4—C16—O17	−8.4 (5)
C16—C4—C5—C6	−179.65 (19)	C3—C4—C16—O18	−8.6 (4)
C4—C5—C6—C1	−0.9 (2)	C5—C4—C16—O18	170.4 (3)
C2—C1—C6—C5	1.6 (2)	O17—C16—O18—C19	2.1 (6)
C7—C1—C6—C5	−179.05 (14)	C4—C16—O18—C19	−176.8 (3)
C2—C1—C7—O8	6.05 (19)	C16—O18—C19—C20	163.8 (7)
C6—C1—C7—O8	−173.30 (12)	C3—C4—C16A—O17A	179.4 (10)
C2—C1—C7—C9	−115.16 (15)	C5—C4—C16A—O17A	1.4 (17)
C6—C1—C7—C9	65.49 (17)	C3—C4—C16A—O18A	1.8 (14)
O8—C7—C9—N15	71.81 (13)	C5—C4—C16A—O18A	−176.2 (8)
C1—C7—C9—N15	−163.95 (11)	O17A—C16A—O18A—C19A	5.2 (19)
O8—C7—C9—C10	−46.57 (14)	C4—C16A—O18A—C19A	−177.5 (10)
C1—C7—C9—C10	77.66 (14)	C16A—O18A—C19A—C20A	79.2 (19)
N15—C9—C10—O11	0.38 (18)		

### 1-Ethoxy-3-[4-(ethoxycarbonyl)phenyl]-3-hydroxy-1-oxopropan-2-aminium chloride Hydrogen-bond geometry (Å, º)

**Table:**

*D*—H···*A*	*D*—H	H···*A*	*D*···*A*	*D*—H···*A*
C7—H7···Cl1	1.00	2.89	3.4784 (13)	119
C7—H7···Cl1^i^	1.00	2.96	3.6365 (14)	126
O8—H8···Cl1	0.84 (2)	2.24 (2)	3.0625 (11)	165.1 (19)
N15—H15*A*···Cl1^i^	0.87 (2)	2.53 (2)	3.2470 (13)	140.7 (16)
N15—H15*A*···O11^ii^	0.87 (2)	2.279 (19)	2.8484 (15)	123.0 (16)
N15—H15*B*···Cl1^iii^	0.94 (2)	2.31 (2)	3.2011 (12)	159.3 (15)
N15—H15*C*···Cl1^iv^	0.94 (2)	2.19 (2)	3.1155 (13)	168.4 (16)
C20—H20*A*···O17^v^	0.98	2.64	3.520 (6)	149
C20*A*—H20*D*···O18*A*^vi^	0.98	2.03	2.75 (2)	129

Symmetry codes: (i) *x*, −*y*+3/2, *z*−1/2; (ii) −*x*, *y*+1/2, −*z*+1/2; (iii) −*x*, −*y*+1, −*z*+1; (iv) −*x*, *y*−1/2, −*z*+1/2; (v) *x*, −*y*+3/2, *z*+1/2; (vi) −*x*+1, *y*+1/2, −*z*+1/2.

## References

[bb1] Ebel, F. & Deuschel, W. (1956). *Chem. Ber.* **89**, 2799–2807.

[bb2] Korsager, S., Nielsen, D. U., Taaning, R. H. & Skrydstrup, T. (2013). *Angew. Chem. Int. Ed.* **52**, 9763–9766. 10.1002/anie.201304072 23881598

[bb3] Sheldrick, G. M. (2015*a*). *Acta Cryst.* A**71**, 3–8.

[bb4] Sheldrick, G. M. (2015*b*). *Acta Cryst.* C**71**, 3–8.

[bb5] Spek, A. L. (2020). *Acta Cryst.* E**76**, 1–11. 10.1107/S2056989019016244 PMC6944088 31921444

[bb6] Stoe & Cie (2020). *X-RED* and *X-AREA*. Stoe & Cie, Darmstadt,

